# Identification of metabolism-associated genes and construction of a prognostic signature in bladder cancer

**DOI:** 10.1186/s12935-020-01627-8

**Published:** 2020-11-04

**Authors:** Chengquan Shen, Jing Liu, Liping Wang, Zhijuan Liang, Haitao Niu, Yonghua Wang

**Affiliations:** 1 Department of Urology, The Affiliated Hospital of Qingdao University, Qingdao, 266000 Shandong People’s Republic of China; 2 Department of Research Management and International Cooperation, The Affiliated Hospital of Qingdao University, Qingdao, Shandong China; 3 Key Laboratory of Urology and Andrology, The Affiliated Hospital of Qingdao University, Qingdao, Shandong China

**Keywords:** Bladder cancer, Metabolism, TGF-β1, Prognosis, TCGA

## Abstract

**Background:**

Bladder cancer (BC) is a commonly diagnosed malignant tumor in the urinary system, with a high morbidity and a high recurrence rate. Current studies indicated that metabolism-associated genes (MAGs) having critical roles in the etiology of BC. The present study aims to identify differentially expressed MAGs and construct a MAGs based prognostic risk signature for BC by using The Cancer Genome Atlas (TCGA) database and proteomics data.

**Methods:**

RNA-sequence data from the TCGA database and proteomics data from our BC samples were used to identify differentially expressed MAGs and construct a MAGs based prognostic signature in BC. Subsequently, survival analysis and nomogram were used to evaluate the prognostic and predictive value of the MAGs based signature in BC. RNA isolation and reverse transcription‑quantitative PCR (RT-qPCR) were further performed to investigate the expression levels of MAGs in BC cells and explore the relationship between MAGs and M2 tumor associated macrophages (TAMs) secreted transforming growth factor-β1 (TGF-β1) in BC cells.

**Results:**

A total of 23 differentially expressed MAGs were identified and five MAGs were finally used to construct a MAGs based signature. Survival analysis revealed that the MAGs based signature was closely correlated with the survival outcomes of patients with BC. A nomogram with the MAGs based signature risk score and clinical features was also constructed to facilitate the individualized prediction of BC patients. RT-qPCR showed that five MAGs were significantly differentially expressed and the expression levels of three MAGs were positively correlated with M2 TAMs secreted TGF-β1 in T24 cells.

**Conclusions:**

Our study identified novel prognostic MAGs and constructed a MAGs based signature, which can be used as an independent factor in evaluating the prognosis of patients with BC. Furthermore, M2 TAMs may promote the expression of MAGs via the TGF-β1 signaling pathway in the microenvironment of BC. Further clinical trials and experimental explorations are needed to validate our observations in BC.

## Background

Bladder cancer (BC) is one of the most malignant and highly aggressive tumors in the urinary system with high recurrence and mortality rates [1]. The 2015 China cancer statistics showed that BC has led to about 80,500 new cases and 32,900 deaths [2]. BC can be generally divided into a non-muscle-invasive and a muscle-invasive disease that is prone to metastasis, based on histological differentiation from normal bladder cells [3]. In the past few decades, numerous efforts have been made to develop diagnostic tools and treatments of BC, but the recurrence and mortality rates of BC are still high. Therefore, novel, early diagnosis, and druggable biomarkers for BC need to be promptly explored.

Recently, metabolic reprogramming has been considered a novel hallmark of cancer cells [4]. The reprogramming of cellular metabolism seems to serve as a vital role in carcinogenesis [5]. As solid tumors usually confronted adverse microenvironments where oxygen and nutrition were limited, the metabolic reprogramming can supply bioenergetic and biosynthetic demands of continuous cancer cell proliferation, including the high uptake of glucose and its use through glycolysis along with increased lipid, nucleotide, and amino acid (AA) biosynthesis [6, 7]. Previous studies also indicated that the alteration of metabolic pathways, such as increased glycolysis under normoxic conditions (Warburg effect), glutamine metabolism, and lipid metabolism, are tightly associated with the occurrence and progression of BC, thus indicating the major roles of metabolic landscapes in BC [8–10]. Metabolic parameters, especially metabolism associated genes (MAGs), has gained great value, power, and importance for cancer research, not only as potential biomarkers in early diagnosis, but also as valuable factors for the discovery of novel mechanisms controlling tumorigenesis, thus paving the way to new treatment strategies and therapies [11–13]. The effect of the metabolism on the progression of the tumor has recently emerged as a new field in BC research. Several metabolites such as taurine, carnitine, and cholinergic compounds have been proposed as biomarkers for BC in urine [14–16]. However, potential reliable and valuable MAGs to early prediction, progression, and the management of BC still limited, and it needs to be further expanded.

In this study, RNA-sequencing (RNA-seq) data from the TCGA database and proteomics data from our samples were used to identify differentially expressed MAGs in BC patients and constructed a MAGs based signature, which was significantly related to the prognosis of BC. A prognostic nomogram combined with the MAGs based signature and clinical characteristics was developed to evaluate the clinical predictive value of the MAGs based signature. In addition, the mRNA expression levels and potential mechanisms of five MAGs in BC cells were further investigated in vitro experiments.

## Methods and materials

### Data collection

RNA-seq expression profiles and clinical information of BC patients were downloaded from the TCGA database (https://tcga‐data.nci.nih.gov/tcga/). The TCGA cohort contained 414 BC patients, and more detailed clinical characteristics are described in Table 1. The MAGs were collected from the Molecular Signature Database v5.1 (MSigDB).

**Table 1:** TCGA BC patient characteristics

Clinical characteristics	Total (414)	%
Age at diagnosis	69 (34–90)	
Gender		
Female	109	26.33
Male	305	73.67
Histologic grade		
High Grade	388	93.71
Low Grade	21	5.07
Stage		
I	2	0.48
II	131	31.64
III	141	34.06
IV	136	32.85
T		
T0	1	0.24
T1	3	0.72
T2	120	28.98
T3	196	47.34
T4	59	14.25
M		
M0	196	47.34
M1	11	2.66
N		
N0	239	57.73
N1	47	11.35
N2	76	18.36
N3	8	1.93

### LC–MS/MS analysis

In the present study, all of the tissue samples were collected from the 10 patients treated with surgical resection, including 10 BC tissues and corresponding normal tissues. The 10 patients who underwent laparoscopic radical cystectomy and who did not receive preoperative radiotherapy and chemotherapy. According to the ethical guidelines as required by the Declaration of Helsinki, informed consent was provided by each patient, and the research protocol was approved by the Ethical Committee of the Affiliated Hospital of Qingdao University.

Comparative proteomic profiling is commonly used to LC–MS/MS. In this study, the same method was performed to characterize the variety of proteins in BC samples and normal samples. The process contained protein extraction, trypsin digestion, TMT/iTRAQ Labeling, HPLC Fractionation, LC–MS/MS Analysis, Database Search, and bioinformatic methods. The enrichment of the differentially expressed protein against all identified proteins was detected by two-tailed Fisher’s exact test, and protein domains with a corrected *p* value < 0.05 were recognized as statistically significant. To further distinguish up- or down-regulated of these proteins in BC, we set the threshold of the ratio to 1.2.

### Identification of differentially expressed MAGs

The differentially expressed MAGs in the TCGA BC cohort were identified using R software (version R 3.5.1, https://bioconductor.org/packages/release/bioc/) [7]. False discovery rate (FDR) < 0.05 and |log2 fold change (FC)| > 1 were recognized as the cutoff values. We applied volcano plots to exhibit significant differentially expressed MAGs in the TCGA BC cohort. Subsequently, the protein levels of these differentially expressed MAGs were further explored in our BC samples. Boxplot was applied to display MAGs, which were differentially expressed in both mRNA and protein levels. Twenty-three MAGs were put into the STRING (Search Tool for the Retrieval of Interacting Gene, https://string-db.org/) to construct a protein–protein interaction (PPI) network [17]. Cytosacpe, an open-source bioinformatics software platform for visualizing molecular interaction networks, was used to screen the PPI network and identify a hub gene in the PPI network. Gene ontology (GO) enrichment analysis and KEGG pathway analysis for differentially expressed MAGs were also performed using the “clusterProfiler” R package. The significantly enriched GO terms and enriched KEGG pathways in MAGs comparing to the genome background were defined by the hypergeometric test. The results with an adjusted p-value < 0.05 were considered as statistically significant.

### Construction and evaluation of a MAGs based signature

Univariate Cox regression analysis was performed using the R package “survival” and genes with a significance level of *p *< 0.05 were selected as candidate prognostic MAGs to establish a signature. The hazard ratios (HRs) were used to identify risk-related MAGs (HR > 1) and protective MAGs (HR < 1). Multivariate Cox regression analysis was utilized to further establish a MAGs based signature to predict the prognosis of BC patients [18]. The prognostic risk score for each BC patient was calculated as follows: (Coefficient gene 1 × expression of gene 1) + (Coefficient gene 2 × expression of gene 2) + ···+(Coefficient gene 5 × expression of gene 5). After that, we classified 403 patients into high- and low-risk groups according to the median value of risk score. Kaplan–Meier analysis was used to estimate the significant differences in survival between the high- and low-risk groups. The survival ROC package was used to conduct the receiver operating characteristic curve (ROC). Univariate and multivariate Cox regression analyses were used to assess prognostic significances of the signature and clinical characteristics. In addition, the Wilcoxon signed-rank test was performed to identify the relationship between the MAGs based signature risk score and clinical characteristics. Moreover, 365 muscle-invasive BC patients (T2–T4) (MIBC) were clustered into two molecular subtypes (basal and luminal) based on gene expression [19]. The differentially expressed levels of five MAGs in two molecular subtypes were analyzed by using the Wilcoxon signed-rank test.

### Development of a nomogram based on the MAGs signature and clinical characteristics

Nomogram is applied to predict the survival outcomes of cancer patients and could dynamically monitor the prognosis of patients. Clinical parameters and the MAGs signature risk score were used to establish a nomogram to evaluate the probability of 1-, 2-, and 3- year OS for BC patients via the R package (https://cran.r-project.org/web/packages/rms/) [20].

### Correlation analysis between risk score and immune cell infiltration in BC

Previous studies showed the immune microenvironment of BC, especially immune cell infiltration in tumors, can influence the metabolic levels of BC to promote or inhibit the progression of BC. Thus, Tumor Immune Estimation Resource (TIMER), a useful resource for comprehensive analysis of tumor-infiltrating immune cells, was employed to explore the correlations between the signature risk score and immune cell infiltration. The composition of six tumor-infiltrating immune cells subsets (B cells, CD4+ T cells, CD8+ T cells, macrophages, neutrophils, and dendritic cells) was estimate by using the TIMER algorithm. The levels of immune cell infiltration in BC patients were obtained from the TIMER website and the relationship between the signature risk score and six tumor-infiltrating immune cells was performed in R.

### RNA isolation and reverse transcription‑quantitative PCR

To further validate the mRNA expression levels of five MAGs in BC cell lines, RNA isolation and reverse transcription‑quantitative PCR (RT-qPCR) were performed. The T24 (human bladder cancer cells) and SV-HUC-1 (human bladder cell biochemistry Pillon) cell lines were supplied by the cell bank of the Chinese Academy of Sciences. The materials used for the cell culture, including the 1640 culture medium, FBS, trypsin, penicillin, and streptomycin, were purchased from Gibco Co. (Grand Island, NY, USA). The total RNA was extracted using Trizol (Takara, code no 9109) according to the manufacturer’s recommendations. For the detection of mRNA levels, the total RNA (500 ng) was transcribed into cDNA using a PrimeScript™ RT reagent kit (Perfect Real Time) (Takara, code no RR037A). All the primers were synthesized by Huada Gene (Beijing, China) and the sequences are shown in Table 2. The amplification of cDNAs was conducted with Roche Light Cycler 480II real-time PCR detection system (Roche, Basel, Switzerland). Gene expression was normalized against β-actin and relative expression levels of *PLOD1*, *CKB*, *PYGB*, *AKR1B1*, and *PDE5A* were determined by the comparative threshold cycle (Ct) method using the formula 2−(ΔΔCt).

**Table 2:** Sequences of the primers used for real-time quantitative PCR

Name of primer	Sequence of primer (5′ to 3′)
PLOD1-F	AAGCCGGAGGACAACCTTTTA
PLOD1-R	GCGAAGAGAATGACCAGATCC
CKB-F	GCTGCGACTTCAGAAGCGA
CKB-R	GGCATGAGGTCGTCGATGG
PYGB-F	AGGTGCGGAAGAGCTTCAAC
PYGB-R	TCGCGCTCGTAGTAGTGCT
AKR1B1-F	TTTTCCCATTGGATGAGTCGG
AKR1B1-R	CCTGGAGATGGTTGAAGTTGG
PDE5A-F	GCAGAGTCCTCGTGCAGATAA
PDE5A-R	GTCTAAGAGGCCGGTCAAATTC

### The relationship between M2 TAMs secreted TGF-β1 and five MAGs in T24 cells

TIMER analysis indicated that the signature was significantly related to macrophages in BC. Current studies have demonstrated that M2 TAMs can secret TGF-β1, which played an important role in the metabolic programming of the microenvironment of tumors. Therefore, the relationship between M2 TAMs secreted TGF-β1 and five MAGs expression levels were further explored in T24 cell lines. The T24 cells were seeded at 2–10 × 10^5^ cells/well in 24-well plates for 24 h and incubated at 37 °C in a humidified atmosphere containing 5% CO2. THP-1 is a human leukemia monocytic cell line, which has been extensively used to study macrophages functions, mechanisms, and signaling pathways [21]. In the present study, THP-1 monocytes were seeded at 2 × 10^5^ cells/well in 24-well plates and were stimulated for 48 h with 100 ng/ml PMA (phorbol-12-myristate-13-acetate) to fully differentiate into macrophages. After that, PMA-differentiated macrophages (M0) were primed with fresh medium supplemented with 20 ng/ml IL-4 for 24 h to the M2 phenotype. Subsequently, M2 TAMs in 24-well plates were cultured with normal 1640 medium (glucose 2000 mg/ml) or 1ug/ml TGF-β1 antibody (AF-246-NA, Bio-Techne China Co. Ltd.) with 24 h. Commercially human TGF-β1 enzyme-linked immunosorbent assay (ELISA) kit (ab100647; Abcam, Cambridge, UK) was used to measure TGF-β1 in the supernatant of cell cultures according to the manufacturer’s instructions. Then the T24 cells were stimulated with the supernatant of M2 TAMs for 48 h. Furthermore, the cells in 24-well plates were stimulated with 0.2 ng/ml recombinant human TGF-beta 1 for 48 h (Cat. No. 7754-BH-005, R&D) to observe the expression of five MAGs in T24 cells. The expression levels of five MAGs were investigated by performing RT-qPCR.

## Results

### Identification of differentially expressed MAGs in BC

**Fig. 1:**
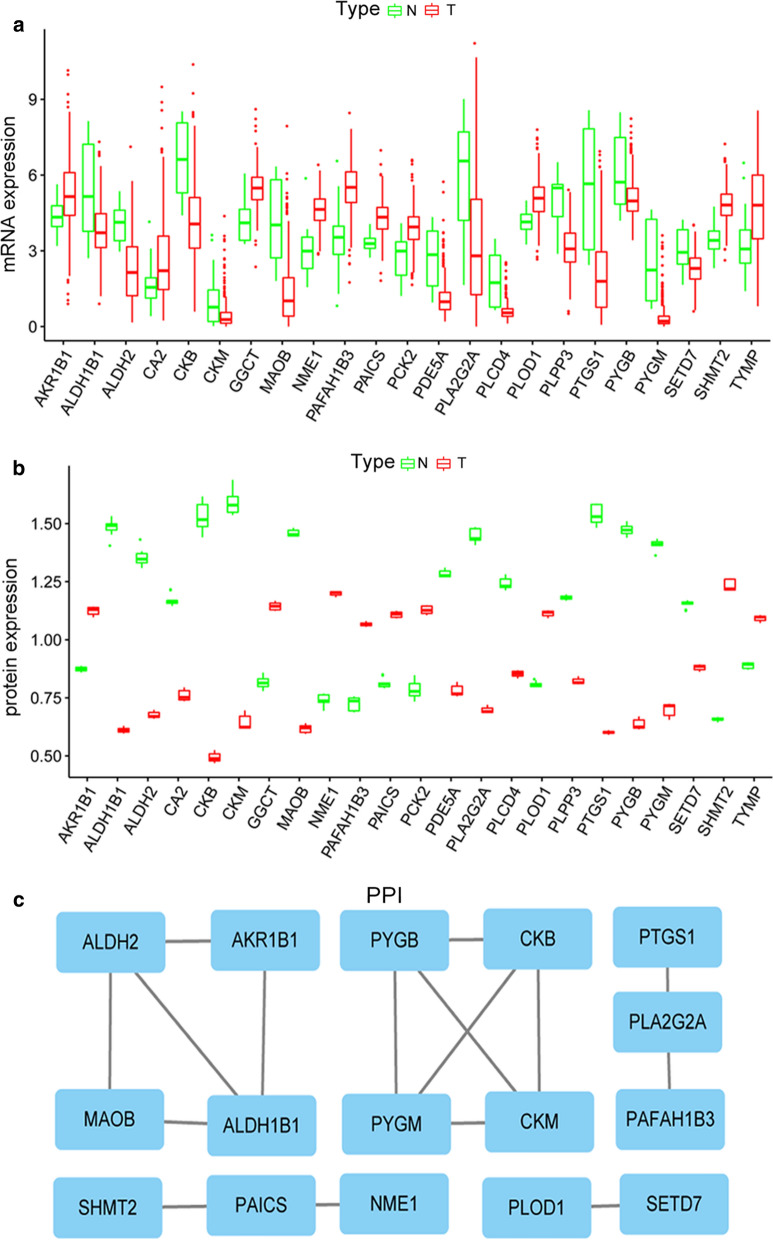
Identification of differentially expressed MAGs and a hub MAGs in BC. **a** Boxplot of the mRNA levels of 23 MAGs in the TCGA BC cohort. **b** Boxplot of protein levels of 23 MAGs in our BC tissue samples. **c** A PPI network of 23 MAGs was constructed and *AKR1B1* was a hub gene in the interaction network by MCODE analysis in Cytoscape

**Fig. 2:**
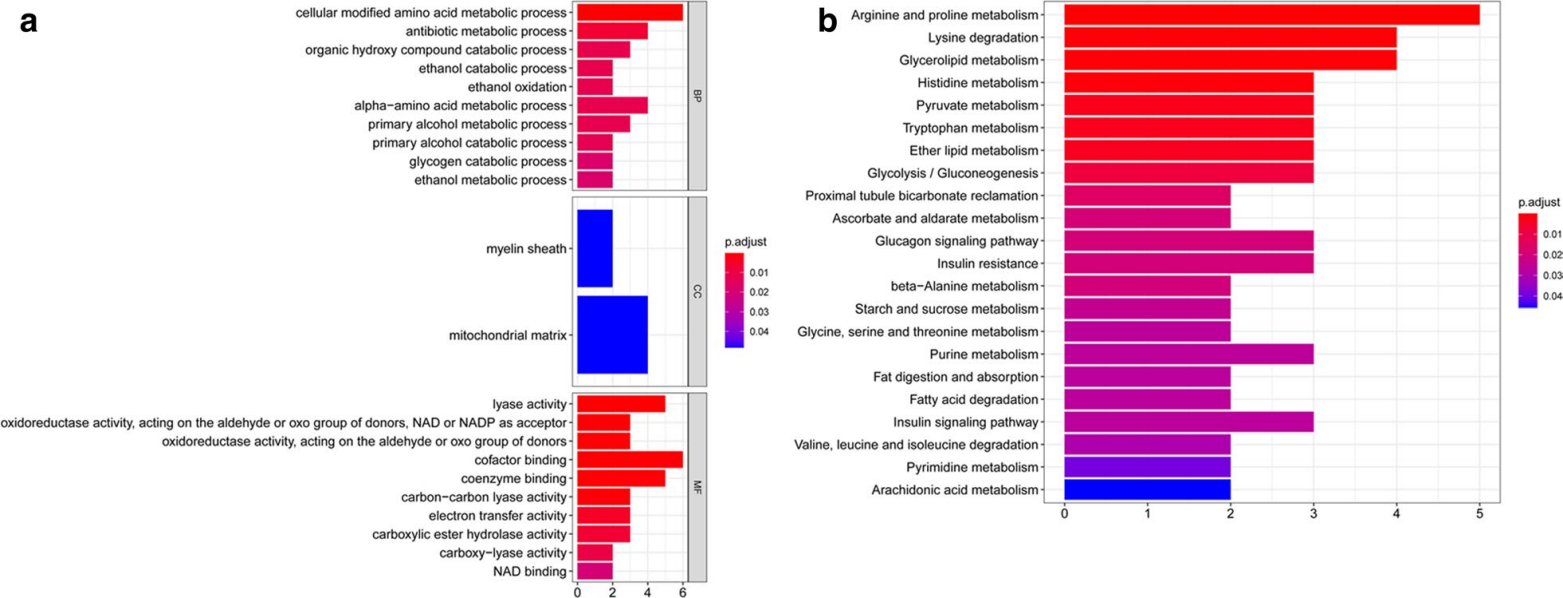
GO and KEGG analyses of differentially expressed MAGs. **a** Heatmap exhibited the enriched GO terms across the differentially expressed MAGs. **b** Heatmap exhibited the enriched KEGG pathways across the differentially expressed MAGs

The mRNA expression data of TCGA BC patients were subjected to identify differentially expressed MAGs. We identified 168 differentially expressed MAGs (68 downregulated and 100 upregulated) between 414 BC tissues and 19 normal tissues. To investigate the differences in the protein levels of these 168 MAGs between BC and normal samples, we collected samples from 10 patients in the Affiliated Hospital of Qingdao University. These samples were processed and analyzed using LC–MS/MS following the process outlined in Additional file 1. The result showed that the protein levels of 23 MAGs were differentially expressed in BC among these 168 MAGs. Boxplots were used to screen the mRNA and protein levels 23 MAGs in BC (Fig. 1a, b). The detailed information of the protein expression of 23 MAGs in our samples was shown in Additional file 2. In addition, a PPI network of 23 MAGs was retrieved from the STRING database and their correlations were further screened in Cytoscape. As a result, we found AKR1B1 was a hub gene in the interaction network by MCODE analysis in Cytoscape (Fig. 1c). To identify the potential mechanisms of these 23 differentially expressed MAGs in BC, we performed GO and KEGG analyses. We found that the most significant GO enriched terms involved in metabolism were amino acid metabolic process, antibiotic metabolic process, organic hydroxy compound catabolic process, alcohol metabolic process, and glycogen catabolic process (BP, biological process); myelin sheath and mitochondrial matrix (CC, cellular component); and lyase activity, oxidoreductase activity cofactor binding, coenzyme binding electron transfer activity, and NAD binding (MF, molecular function) (Fig. 2a). In the KEGG enrichment analysis, these MAGs were primarily correlated with pathways related to arginine and proline metabolism, lysine degradation, glycerolipid metabolism, histidine metabolism, pyruvate metabolism, tryptophan metabolism, ether lipid metabolism, and glycolysis (Fig. 2b).

### Identification of prognosis-related MAGs and construction of a MAGs based prognostic signature

**Fig. 3:**
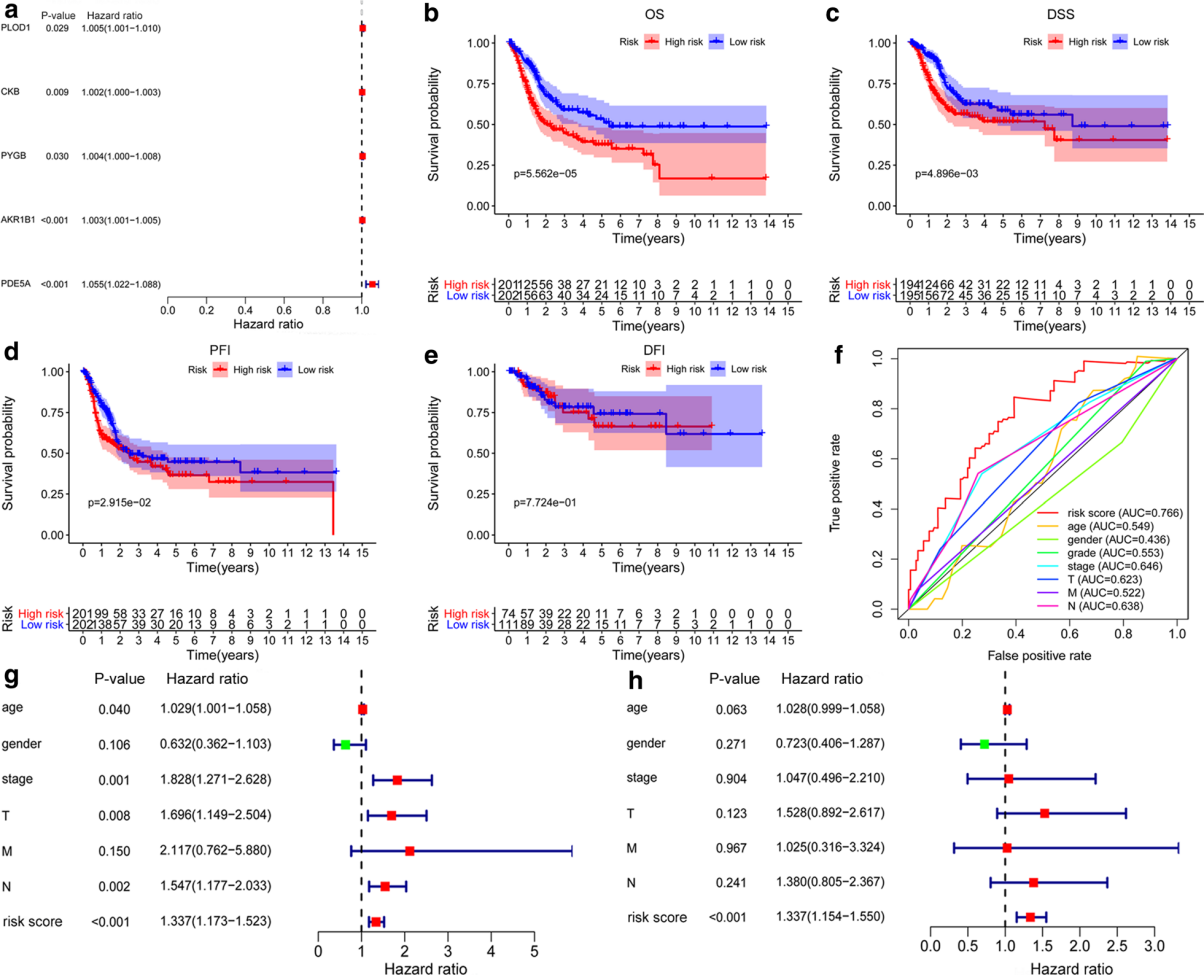
Construction of a MAGs based signature to predict the prognosis of BC. **a** Univariate Cox regression analysis showed that five MAGs are closely associated with the OS of BC patients. **b–d** Kaplan–Meier curves revealed that the high-risk group had significantly shorter overall survival (OS), disease-specific survival (DSS), and progression-free interval (PFI) compared with the low-risk group. **e** However, the signature was not associated with disease-free interval (DFI). **f** ROC curves showed that the area under the curves (AUCs) of the risk score, age, gender, grade, stage, T, M, and N were 0.766, 0.549, 0.436, 0.553, 0.646, 0.623, 0.522, and 0.638. **g** Univariate Cox regression analysis showed that age, stage, T, N, and risk score were significantly related to the survival of BC patients. **h** Multivariate Cox regression analysis demonstrated that the signature could serve as an independent prognostic predictor for BC patients

By performing univariate Cox regression analysis on 23 MAGs, a total of 5 MAGs were identified to have significant prognostic value in BC (*P *< 0.05) (Fig. 3a). Five MAGs were considered as risk factors with HR values greater than 1. Subsequently, we utilized multivariate Cox regression analysis to construct a prognostic signature, which contained five MAGs, including *PLOD1* (procollagen-lysine,2-oxoglutarate 5-dioxygenase 1), *CKB* (creatine kinase B), *PYGB* (glycogen phosphorylase B), *AKR1B1* (aldo–keto reductase family 1 member B), and *PDE5A* (phosphodiesterase 5A) (Table 3). We further calculated the prognostic risk score for each BC patient as follows: risk score = (0.0049 × expression level of *PLOD1*) + (0.0018 × expression level of *CKB*) + (0.0033 × expression level of *PYGB*) + (0.0031 × expression level of *AKR1B1*) + (0.0486 × expression level of *PDE5A*). Four hundred and three BC patients were subdivided into high-risk and low-risk groups according to the median value of risk score. K-M survival curve analysis showed that the MAGs based signature was closely associated with poor overall survival (OS) (*P *= 5.562e-05), disease-specific survival (DSS) (*P *= 4.896e−03), and progression-free interval (PFI) (*P *= 2.915e−02) in BC (Fig. 3b–d). However, the signature risk score was not correlated with the disease-free interval (DFI) (*P *= 7.724e−01) of BC patients (Fig. 3e). ROC curve analysis was used to further measure the predictive performance of the MAGs based signature risk score. The area under the curves (AUCs) for the MAGs signature, age, gender, grade, stage, T, M, N were 0.766, 0.549, 0.436, 0.553, 0.648, 0.623, 0.522, and 0.638, which indicated superior predictive accuracy of the MAGs signature risk score in survival outcomes (Fig. 3f). We further used univariate and multivariate Cox regression analyses to assess the prognostic values of the MAGs based signature and clinical features. Univariate Cox regression analysis showed that age, stage, T (tumor), N (node), and risk score were related to the survival of BC patients (Fig. 3g). Subsequently, multivariate Cox regression analysis indicated that the MAGs based signature was an independent prognostic factor for BC (*P *< 0.001, Fig. 3h).

**Table 3:** Multivariate Cox regression analysis for OS of five MAGs in BC

Gene name	coef	HR	HR.95L	HR.95H	*P*-value
*PLOD1*	0.0049	1.0049	0.9999	1.0099	0.0509
*CKB*	0.0018	1.0018	1.0003	1.0032	0.0173
*PYGB*	0.0033	1.0033	0.9991	1.0075	0.1259
*AKR1B1*	0.0031	1.0031	1.0015	1.0046	0.0001
*PDE5A*	0.0486	1.0498	1.0145	1.0865	0.0054

*HR* hazard ratio

### Association between the MAGs signature risk score and clinicopathologic characteristics

**Fig. 4:**
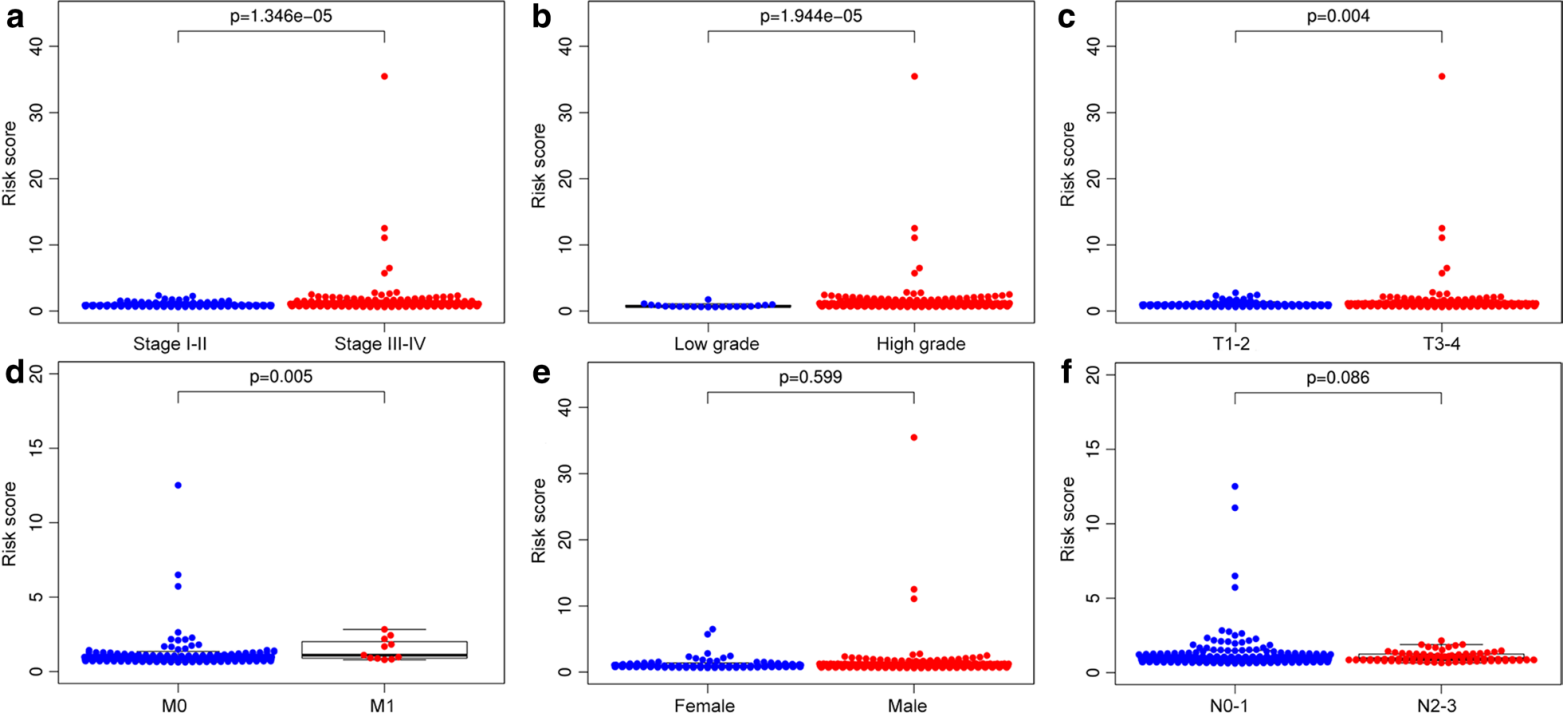
The relationship between the MAGs signature risk score and clinicopathological characteristics. **a–d** The MAGs signature risk score was associated with the stage, grade, T, and M of BC. **e**, **f** However, the risk score was not related to the gender and N of BC

**Fig. 5:**
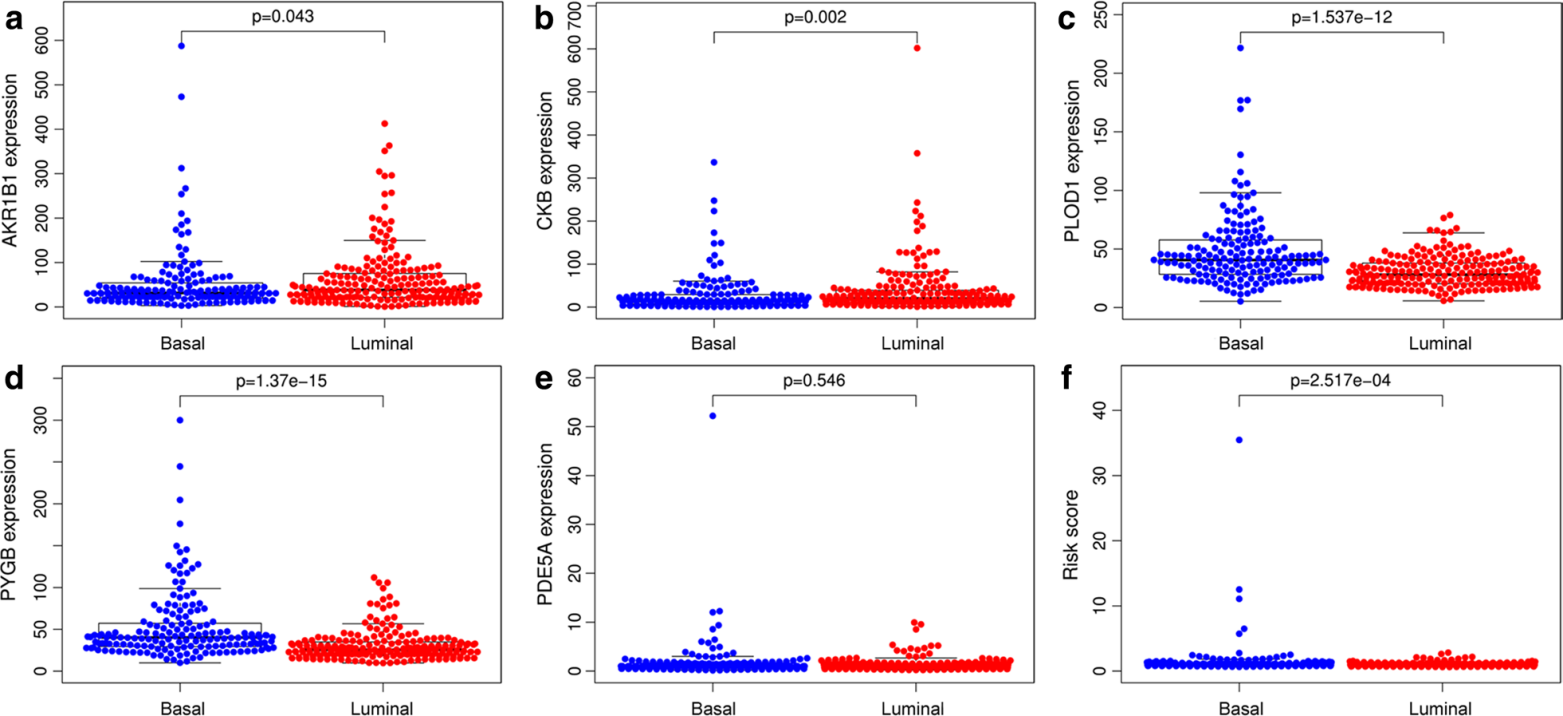
The expression level of five MAGs in different molecular subtypes of BC. **a–d** *AKR1B1*, *PLOD1*, and *PYGB* were highly expressed in the basal subtype and *CKB* was highly expressed in the luminal subtype. **e** However, the expression of *PDE5A* was not associated with the molecular subtypes of BC. **f** Furthermore, MIBC patients with high MAGs based signature risk score may indicate the feature of a basal subtype

The treatment methods for BC patients depend largely on clinical characteristics, and we evaluated whether there was a statistically significant difference between risk score and clinicopathological characteristics. Our study revealed that the MAGs based signature risk score was correlated with the stage (*P *= 1.346e−05), grade (*P *= 1.944e−05), T (*P *= 0.004), M (metastasis) (*P *= 0.005) of BC patients (Fig. 4a–d). However, the risk score was not related to gender (*P *= 0.599) and N (*P *= 0.086) of BC patients (Fig. 4e, f). In recent years, several independent studies have shown that BC has distinct molecular subtypes, which were associated with different outcomes of BC patients [22–24]. Therefore, the expression levels of five MAGs in two molecular types were further analyzed. The results indicated that *AKR1B1*, *PLOD1*, and *PYGB* were highly expressed in the basal subtype and *CKB* was highly expressed in the luminal subtype (Fig. 5a–d). However, the expression of *PDE5A* was not associated with the molecular subtypes of BC (Fig. 5e). Furthermore, MIBC patients with high MAGs based signature risk score may indicate the features of a basal subtype (Fig. 5f).

### Construction of a prognostic nomogram for BC

**Fig. 6:**
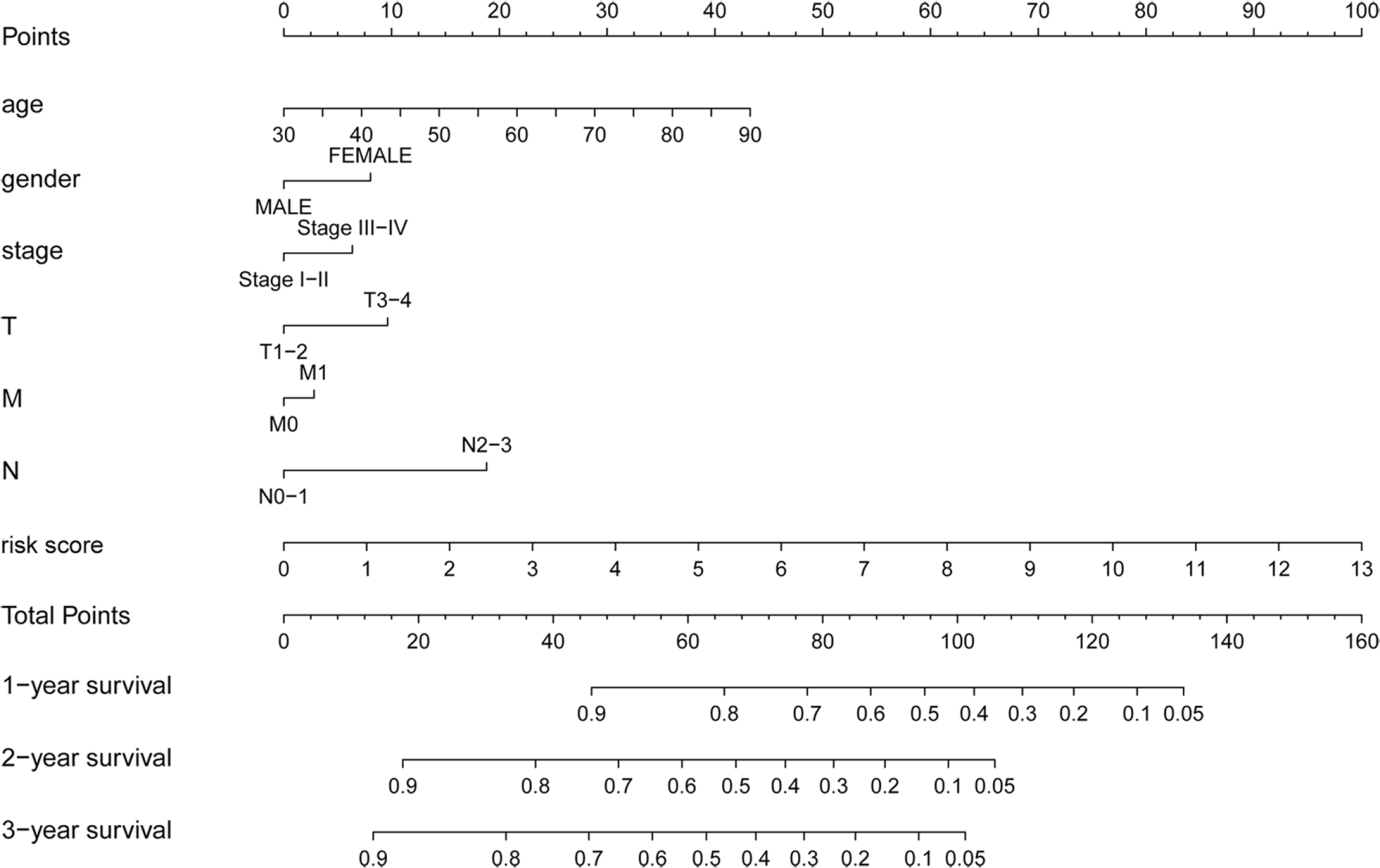
A nomogram based on the MAGs signature and clinical characteristics was established for BC patients. The nomogram could superiorly predict 1-, 2-, and 3-year OS of BC patients

To establish a clinically applicable method for monitoring the prognosis of BC patients, we generated a nomogram to predict the survival of BC patients, by combining age, gender, grade, stage, T, N, M with the MAGs based signature risk score. The result showed that the prognostic nomogram could superiorly predict the 1-, 2-, and 3-year survival outcomes of BC patients (Fig. 6).

### Correlation analysis between the risk score and immune cell infiltration in BC

To identify the significance of the MAGs based signature in the tumor microenvironment, the relationship between the abundance of six types of tumor‐infiltrating immune cells (B cells, CD4+ T cells, CD8+ T cells, neutrophils, macrophages, and dendritic cells) and the MAGs based signature risk score was explored in BC. The results indicated that the risk score was positively associated with the infiltration of macrophages (*P *= 1.354e−08) and dendritic cells (*P *= 6.016e−04,). However, the risk score was not correlated with the infiltration of B cells, CD4+ T cells, CD8+ T cells, and neutrophils (Table 4).

**Table 4:** Correlation analysis between risk score and immune cell infiltration in BC

Immune cell	Correlation	*P*-value
Macrophage	0.278	1.354e−08
Dendritic	0.170	6.016e−04
B cell	− 0.053	0.287
CD4+ T cell	0.054	0.279
CD8+ T cell	0.065	0.191
Neutrophil	0.037	0.463

### M2 TAMs may promote the expression levels of MAGs via the TGF-β1 signaling pathway

**Fig. 7:**
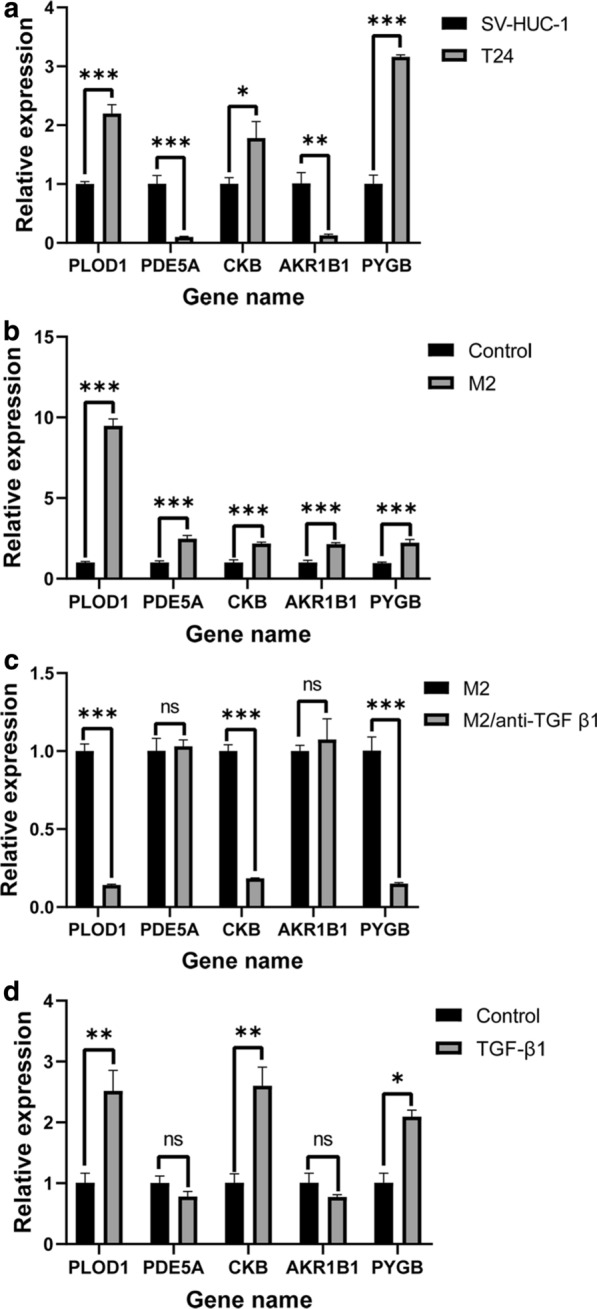
RT-PCR validation of five MAGs expression in BC cells. **a** The significant differences in the expression levels of five MAGs between T24 and SV-HUC-1 cell lines. Among these five MAGs, *PLOD1*, *CKB*, and *PYGB* were upregulated, *PDE5A* and *AKR1B1* were downregulated in T24 cells. **b** Compared with the unstimulated T24 cells, the expression of five MAGs was significantly elevated in T24 cell lines after stimulated with the supernatant of M2 TAMs. **c** The expression levels *PLOD1*, *CKB*, and *PYGB* were significantly downregulated in T24 cells when stimulated with the low TGF-β1 supernatant of M2 TAMs. **d** The expression levels of *PLOD1*, *CKB*, and *PYGB* were elevated in T24 cells after stimulated with TGF-β1. *P < 0.05; **P < 0.005; ***P < 0.0005

RNA isolation and reverse transcription‑quantitative PCR (RT-qPCR) was further performed to validate the expression levels of five selected MAGs in T24 and SV-HUC-1 cell lines. The results demonstrated significant differences in the expression levels of five MAGs between T24 and SV-HUC-1 cell lines (Fig. 7a). Among these five MAGs, *PLOD1*, *CKB*, *PYGB* were upregulated, *PDE5A* and *AKR1B1* were downregulated in T24 cells. Compared with the unstimulated T24 cells, the expression levels of five MAGs were significantly elevated in T24 cell lines after stimulated with the supernatant of M2 TAMs (Fig. 7b). Furthermore, we used the TGF-β1 inhibitor to inhibit the production of TGF-β1 in M2 TAM cells. Compare to M2 TAMs without TGF-β1 inhibitor, the production of TGF-β1 in M2 TAMs with TGF-β1 inhibitor was significantly decreased (Additional file 3). The expression levels *PLOD1*, *CKB*, and *PYGB* were significantly downregulated in T24 cells when stimulated with the low TGF-β1 supernatant of M2 TAMs (Fig. 7c). To investigate whether TGF-β1 alone can affect the expression of MAGs in T24 cells, the T24 cells were stimulated with recombinant human TGF-beta 1. Compared with the unstimulated T24 cells, the expression of *PLOD1*, *CKB*, and *PYGB* was elevated, but the expression of *PDE5A* and *AKR1B1* was not significantly changed (Fig. 7d).

In summary, five MAGs were differentially expressed and M2 TAMs secreted TGF-β1 can promote three MAGs expression in BC cells.

## Discussion

Metabolic alteration in the tumor microenvironment played a vital role in carcinogenesis, progression, and therapeutic resistance of many cancers, especially BC. Previous studies had demonstrated that the alteration of glutamine and glycolytic levels in BC cells could promote the progression of BC [7, 25]. Considering the importance of the metabolic environment in cancer development, it is crucial to identify metabolic-related prognostic biomarkers for BC. In the present study, we identify 23 MAGs, which were differentially expressed in both mRNA and protein levels. Among 5 MAGs, *AKR1B1* was a hub gene, which may be a potential biomarker in BC. The role of *AKR1B1* in BC not clear but growing studies are suggesting to *AKR1B1* have an important impact on cancer progression. *AKR1B1*, a member of the aldo/keto reductase superfamily, was associated with the poor survival outcomes of basal-like breast cancer and can promote the occurrence and metastasis of cancer by activating epithelial-mesenchymal transition [26]. *AKR1B1* was highly expressed in multiple tumors and was associated with nuclear factor kappa‐light‐chain‐enhancer of activated B cells (NFκB), survival proteins and pathways like the mammalian target of rapamycin (mTOR) and protein kinase B (PKB), and other regulatory factors in response to reactive oxygen species (ROS) and prostaglandin synthesis [27]. Furthermore, inhibition of *AKR1B1* could render cancer cells more sensitive to anti‐cancer therapy or alleviate the adverse effects of therapy. Therefore, AKR1B1 could also be considered as a potential biomarker in BC.

Subsequently, five differentially expressed MAGs (*PLOD1*, *CKB*, *PYGB*, *AKR1B1*, *PDE5A*) were finally used to construct a prognostic signature and survival analysis indicated that high MAGs risk scores were significantly related to the poor OS, DSS, and PFI of BC patients. Subsequently, Cox regression analyses indicated that the MAGs signature was an independent prognostic factor for BC patients and closely related to stage, grade, T, and M. Notably, *AKR1B1*, *PLOD1*, and *PYGB* were highly expressed in the basal subtype and CKB was highly expressed in the luminal subtype. Previous studies indicated that these MAGs were significantly associated with multiple metabolic signs of progress in tumors. Thus, four MAGs may be novel biomarkers to predict the metabolic status of the molecular subtype of BC. Nomograms have been used to predict the prognosis of patients by incorporating a variety of significant prognostic factors. We established a prognostic nomogram with clinical factors and the MAGs based signature risk score, which can superiorly predict the OS of BC patients. RT-PCR showed that the expression of five MAGs were elevated in T24 cells and M2 TAMs can promote the expression of *PLOD1*, *CKB*, and *PYGB* in T24 cells by secreting TGF-β1. As previous studies reported, TGF-β, a multifunctional cytokine in cancer, acting as both tumor suppressor and a factor that promotes cancer invasion and metastasis [28–30]. TGF-β binds to its type 2 receptor leading to activating its type 1 receptor, which then phosphorylates intracellular effectors Smad2 and Smad3. The phosphorylated Smad2/3 proteins form a complex with common mediator Smad4 and then translocate into the nucleus to regulate TGF-β target gene expression [31–33]. Emerging evidence has proven that TGF-β signaling possesses both Smad and non-Smad pathways, and regulated tumorigenesis via different molecular mechanisms, including, TGF-β1/Smad2/3, PI3K-AKT-mTOR, Wnt, Notch, and ERK, p38, and JUN N-terminal kinase (JNK) MAPK pathways [34, 35]. Various studies also indicated that TGF-β was significantly associated with the expression of MAGs and metabolic reprogramming of cancer [36–38]. Therefore, M2 TAMs secreted TGF-β1 may influence the metabolic reprogramming in BC by these signaling pathways, especially Smad pathways, to promote the recurrence and progression of BC. However, further experimental exploration is needed to verify our observations and explore the precise role of TGF-β1 in BC.

In addition, *PLOD1* encodes lysyl hydroxylases, which are crucial for collagen biosynthesis, cross‐linking, and deposition and can promote cancer progression and metastasis [39]. Yamada et al. [40] revealed that overexpression of *PLOD1* was closely related to poor survival and downregulation of *PLOD1* can decrease the progression of BC. *CKB* was participated in metabolic processes involving glycolysis and could serve as a biomarker for predicting tumor progression [41]. However, the precise role of *CKB* in the occurrence and progression of BC has not been well studied. *PYGB* is an enzyme that metabolizes glycogen and can influence the growth and apoptosis of the cancer cell by regulating the NF‑κB/Nrf2 signaling pathway [42]. Moreover, *PYGB* was associated with the poor prognosis of cancer and can promote the proliferation and invasion of cancer cells by activating Wnt/β-catenin signaling [18, 43]. *PDE5A* was overexpressed in various tumors and inhibition of *PDE5A* can induce apoptosis and attenuate β-catenin-mediated transcription in breast cancer cells [44–46].

Although some of these MAGs have previously been confirmed as prognostic markers for BC, in this study five MAGs, which were identified closely associated with the survival outcomes of BC by bioinformatics methods, were integrated into a MAGs based signature. In addition, M2 TAM can influence the expression of MAGs in BC cells via TGF-β1 signaling pathway. All these MAGs have participated in the process of metabolic signaling pathways, such as glucose metabolism and lipid metabolism. Therefore, we suggested that the MAGs based signature can also reflect the metabolic status of patients with BC. However, several limitations should be considered in our research. Firstly, this is a retrospective study. Therefore, we could not obtain complete information, which may lead to bias. Secondly, more samples need to be further confirmed before clinical application and further experimental studies are needed to investigate the potential molecular mechanisms of these MAGs in BC.

## Conclusions

In conclusion, our study identified 23 differentially expressed MAGs and established a MAGs based signature, which can be used as an independent signature in evaluating the prognosis of patients with BC. Furthermore, M2 TAMs may promote the expression of *PLOD1*, *CKB*, and *PYGB* via the TGF-β1 signaling pathway. Further clinical trials and experimental exploration are needed to validate our observations in BC.

## Supplementary information

**Additional file 1.** The process of proteomic profiling. The process contained protein extraction, trypsin digestion, TMT/iTRAQ Labeling, HPLC Fractionation, LC–MS/MS Analysis, Database Search, and bioinformatic methods.

**Additional file 2: Table S1.** The differences in the protein levels of MAGs between BC samples and normal samples.

**Additional file 3.** The production of TGF-β1 in M2 TAM cells. Compare to M2 TAM cells without TGF-β1 inhibitor, the production of TGF-β1 in M2 TAM cells with TGF-β1 inhibitor were significantly decreased. *P < 0.05; **P < 0.005; ***P < 0.0005.

## Data Availability

The data used to support the findings of this study is included in the article, and the data are available from the corresponding author upon request.

## Abbreviations

BC: Bladder cancer
MAGs: Metabolism-associated genes
TCGA: The Cancer Genome Atlas
RT-qPCR: RNA isolation and reverse transcription‑quantitative PCR
TAMs: Tumor associated macrophages
TGF-β1: Transforming growth factor-β1
AA: Amino acid
FDR: False discovery rate
FC: Fold change
STRING: Search Tool for the Retrieval of Interacting Gene
GO: Gene ontology
HRs: Hazard ratios
ROC: Receiver operating characteristic
TIMER: Tumor Immune Estimation Resource
BP: Biological process
CC: Cellular component
MF: Molecular function
PLOD1: Procollagen-lysine,2-oxoglutarate 5-dioxygenase 1
CKB: Creatine kinase B
PYGB: Glycogen phosphorylase B
AKR1B1: Aldo-keto reductase family 1 member B
PDE5A: Phosphodiesterase 5A
OS: Overall survival
DSS: Disease-specific survival
PFI: Progression-free interval
DFI: Disease-free interval
AUCs: Area under the curves
mTOR: Mammalian target of rapamycin
PKB: Protein kinase B
ROS: Reactive oxygen species
