# Supplementary figures and images for: Identification of metabolism-associated genes and construction of a prognostic signature in bladder cancer
Source: Cancer Cell Int. 2020 Nov 4;20:538. doi: 10.1186/s12935-020-01627-8 (PMC7643334; doi:10.1186/s12935-020-01627-8)

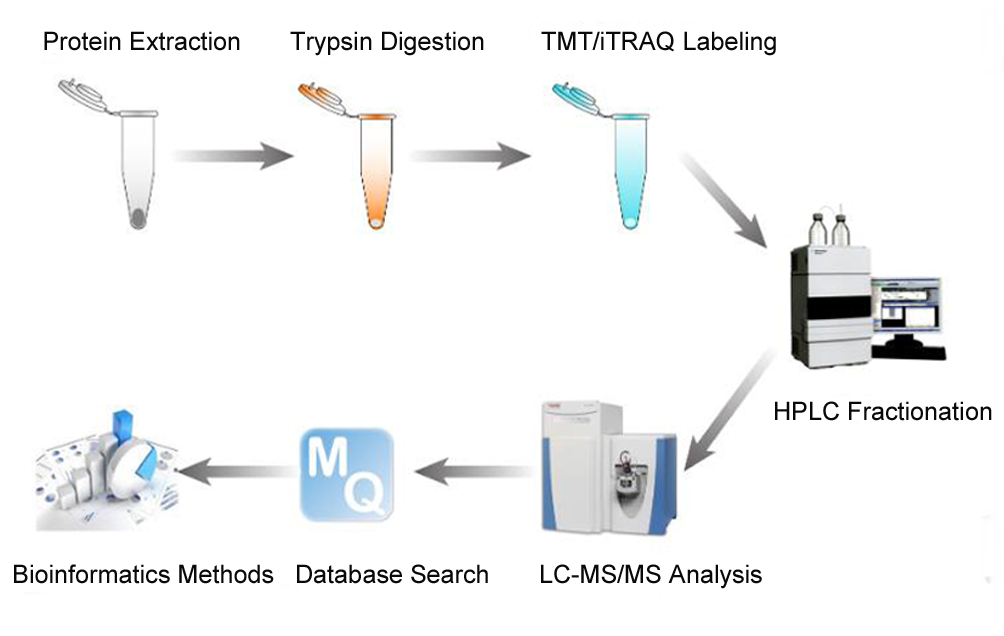

Supplement: Supplementary file 1 — Additional file 1. The process of proteomic profiling. The process contained protein extraction, trypsin digestion, TMT/iTRAQ Labeling, HPLC Fractionation, LC–MS/MS Analysis, Database Search, and bioinformatic methods. [file 12935_2020_1627_MOESM1_ESM.tif]

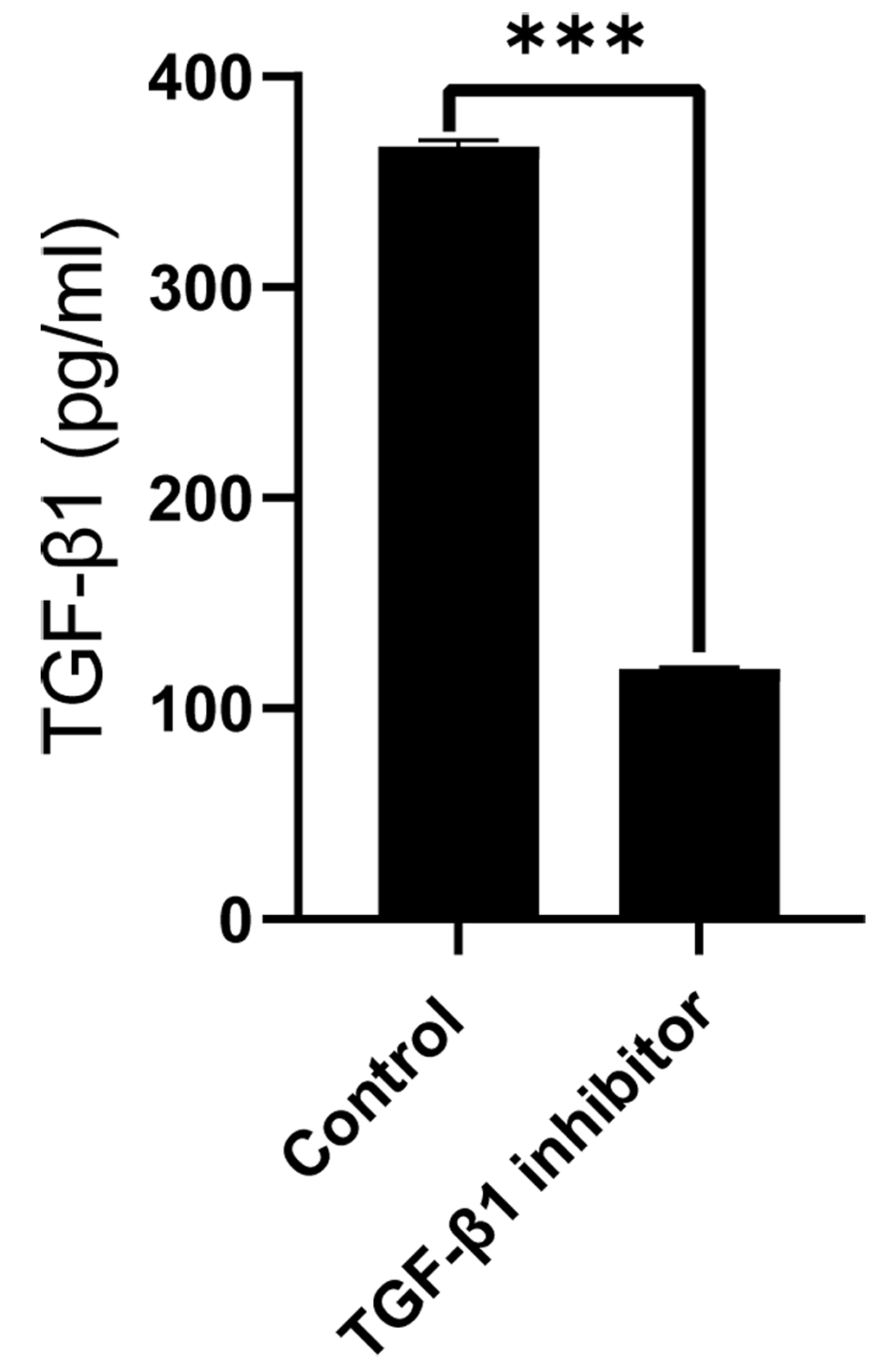

Supplement: Supplementary file 3 — Additional file 3. The production of TGF-β1 in M2 TAM cells. Compare to M2 TAM cells without TGF-β1 inhibitor, the production of TGF-β1 in M2 TAM cells with TGF-β1 inhibitor were significantly decreased. *P < 0.05; **P < 0.005; ***P < 0.0005. [file 12935_2020_1627_MOESM3_ESM.tif]
