# Supplementary material for: Identification of metabolism-associated genes and construction of a prognostic signature in bladder cancer
Source: Cancer Cell Int. 2020 Nov 4;20:538. doi: 10.1186/s12935-020-01627-8 (PMC7643334; doi:10.1186/s12935-020-01627-8)
Supplement: Supplementary file 2 — Additional file 2: Table S1. The differences in the protein levels of MAGs between BC samples and normal samples. [file 12935_2020_1627_MOESM2_ESM.docx]

**Additional file 2: Table S1**  **The differences in the protein levels of MAGs between BC samples and normal samples.**

| Gene name | T/N Ratio | Regulated Type | P-value | Subcellular localization |
| --- | --- | --- | --- | --- |
| AKR1B1 | 1.422 | Up | 5.91E-05 | cytoplasm |
| ALDH1B1 | 0.299 | Down | 2.46E-06 | mitochondria |
| ALDH2 | 0.388 | Down | 2.65E-06 | mitochondria |
| CA2 | 0.566 | Down | 3.52E-05 | cytoplasm |
| CKB | 0.213 | Down | 3.85E-06 | cytoplasm |
| CKM | 0.272 | Down | 3.62E-05 | cytoplasm |
| GGCT | 1.637 | Up | 3.8E-05 | cytoplasm |
| MAOB | 0.306 | Down | 6.09E-07 | cytoplasm |
| NME1 | 1.951 | Up | 4.20E-05 | cytoplasm |
| PAFAH1B3 | 1.66 | Up | 6.48E-05 | extracellular |
| PAICS | 1.557 | Up | 1.22E-06 | cytoplasm |
| PCK2 | 1.693 | Up | 8.07E-05 | mitochondria |
| PDE5A | 0.494 | Down | 2.21E-05 | nucleus |
| PLA2G2A | 0.362 | Down | 2.14E-06 | extracellular |
| PLCD4 | 0.583 | Down | 1.91E-05 | nucleus |
| PLOD1 | 1.567 | Up | 9.62E-07 | extracellular |
| PLPP3 | 0.607 | Down | 4.42E-06 | plasma membrane |
| PTGS1 | 0.275 | Down | 1.33E-06 | extracellular |
| PYGB | 0.314 | Down | 2.89E-06 | cytoplasm |
| PYGM | 0.38 | Down | 1.98E-05 | cytoplasm |
| SETD7 | 0.691 | Down | 2.30E-05 | cytoplasm |
| SHMT2 | 2.329 | Up | 1.47E-06 | mitochondria |
| TYMP | 1.324 | Up | 5.80E-05 | nucleus |
